# Clinical efficacy of nanohydroxyapatite-containing toothpaste at relieving dentin hypersensitivity: an 8 weeks randomized control trial

**DOI:** 10.1038/s41405-021-00080-7

**Published:** 2021-06-25

**Authors:** Bennett Tochukwu Amaechi, Kelly C. Lemke, Shyamali Saha, Minh N. Luong, Jonathan Gelfond

**Affiliations:** 1 Department of Comprehensive Dentistry, University of Texas Health San Antonio, San Antonio, TX USA; 2 Department of Developmental Dentistry, University of Texas Health San Antonio, San Antonio, TX USA; 3 Department of Epidemiology and Biostatistics, University of Texas Health San Antonio, San Antonio, TX USA

**Keywords:** Dental treatments, Gingival recession, Dentine, Tooth brushing

## Abstract

**Objective:**

The objective of this study was to investigate and compare the effectiveness of several toothpastes containing nanohydroxyapatite (nano-HAP) to relieve dentin hypersensitivity (DHS) with that of a commercial desensitizing dentifrice containing calcium sodium phosphosilicate (CSPS).

**Materials and methods:**

In this double-blind, randomized, parallel-group clinical trial, patients diagnosed with DHS and qualified to participate were randomized into four groups: toothpaste containing 10% nano-HAP (10%nano-HAP), 15% nano-HAP (15%nano-HAP), 10% nano-HAP supplemented with potassium nitrate (KNO_3_) (10%nano-HAPKN), or CSPS. Subjects’ baseline and post-treatment sensitivities were assessed using visual analog scale (VAS) after the application of ice-cold and air stimuli. Subjects used their assigned toothpaste for routine toothbrushing twice daily. Post-treatment sensitivity was assessed every 2 or 8 weeks. Mean change in VAS (mm) from baseline at each time point were compared using random-intercept, mixed-model analysis and Duncan test (*P* < 0.05).

**Results:**

With either air or cold stimulus, VAS indicated a significant (*P* < 0.001) reduction from baseline DHS at each time point with all test toothpastes. Among the nano-HAP toothpastes, 15%nano-HAP and 10%nano-HAPKN were consistent in DHS reduction with both stimuli. With either stimuli, the CSPS did not significantly differ from 15%nano-HAP and 10%nano-HAPKN at any time point.

**Conclusions:**

Toothpaste containing nano-HAP (10 or 15%) alone or supplemented with KNO_3_ was as effective as CSPS for relief of DHS symptoms when used at least twice daily.

## Introduction

Dentin hypersensitivity (DHS) is a common clinical condition characterized by transient, sharp pain arising from exposed tooth dentin in response to external stimuli, typically thermal, evaporative, tactile, electrical, osmotic, or chemical, that cannot be attributed to any other dental disease.^1^ DHS is an exaggerated response that occurs when a benign stimulus affects dentin tubules that have become exposed due to gingival recession, loss of enamel, or loss of the cementum layer that covers the root surface.^2^ The pain of DHS leads to physical and psychological discomfort that can range from a minor annoyance to chronic irritation. It has the potential to negatively impact patients’ oral health and quality of life including an adverse effect on the patient’s ability to eat, drink, and maintain good oral hygiene.^3^

DHS prevalence figures from around the globe range from 4 to 74% lifetime incidence of dentinal hypersensitivity; females are more likely to be affected than males.^4–6^ DHS is most often reported in patients aged 20–50 years, with prevalence peaking between 30 and 40 years.^5^ The prevalence of DHS in patients referred for specialist periodontal care is higher than that in the general patient population.^7^ The most commonly affected teeth are the maxillary and mandibular canines and premolars, with the cervical area of the buccal surface being the most affected tooth region.^2^

The most widely held theory for DHS etiology is the hydrodynamic theory, in which pain results from fluids within exposed dentinal tubules being disturbed by external trigger stimuli; rapid fluid movement within the tubules activates sensory nerve receptors within the pulp, resulting in the characteristic short, sharp pain of DHS.^8^

The primary therapeutic approaches to DHS are nerve desensitization via chemical agents that suppress or modify nerve polarization, or occlusion of the dentin tubules to decrease dentin permeability and thereby impede fluid movement within the tubules.^9^ Potassium ions, typically in the form of potassium nitrate (KNO_3_), modify the nerve impulse by depolarizing nerve fiber membranes and rendering them unable to repolarize, resulting in a reduction in tooth sensitivity; however, no strong evidence is available to support the efficacy of KNO_3_-containing dentifrices in treating DHS.^10^

The most common therapeutic approach is the chemical or physical occlusion of the dentin tubules.^9^ Many of the agents in this category result in the formation of either intratubular precipitates or a smear layer over dentin that blocks entry to the tubules. However, the current body of published research has not established a so-called gold standard for tubule occlusion.

Professionally applied fluoride varnish to chemically occlude tubules has been used extensively in the treatment of DHS, and while some studies have shown it to result in significant, rapid improvement in symptoms,^11,12^ two randomized controlled trials showed that the efficacy of 5% sodium fluoride varnish was not significantly different from control varnish.^13,14^ Products containing oxalate compounds^15,16^ or silica-based bioglass, in particular calcium sodium phosphosilicate (CSPS),^17–20^ have demonstrated more promising results. Studies on the efficacy of products containing casein phosphopeptide-amorphous calcium phosphate or strontium chloride have been inconclusive.^21–23^

Nanohydroxyapatite (nano-HAP) is another agent with the potential to treat DHS. Studies have shown that nano-HAP applied as a toothpaste or in topical cream form has the ability to remineralize initial caries lesions.^24–35^ It is thought that nano-HAP may serve as a calcium and phosphate reservoir, maintaining a state of supersaturation of these ions at the tooth surface and thereby promoting crystal deposition and growth.^36^ Initial in vitro studies established the potential of nano-HAP to occlude dentin tubules as a surrogate measure of its ability to relieve DHS symptoms.^37–40^ A further study used an in situ model to demonstrate that nano-HAP-containing toothpastes had the ability to form a protective layer on the surface of dentin in human root specimens, physically occluding dentin tubules as a measure of their potential to treat DHS.^41^ A clinical trial has shown a significant reduction in DHS immediately and at 4 weeks after a single application of 15% nano-HAP paste.^42^

The main objective of the present clinical study, which tested a number of toothpaste products, was to investigate the effectiveness of several nano-HAP-containing toothpastes produced by Sangi Co., Ltd of Japan to relieve DHS, compared with that of a commercial desensitizing dentifrice-containing CSPS. This study tested the hypothesis that nano-HAP-containing toothpastes are as effective as a CSPS-containing toothpaste (Sensodyne™ Repair & Protect with NovaMin^®^; GlaxoSmithkline Consumer Healthcare, South Africa) in relieving DHS.

## Materials and methods

This was a double-blind, randomized, parallel-group, single-center clinical trial to test the ability of toothpaste containing varying percentages of nano-HAP to relieve the symptoms of DHS. The primary outcome to be examined was the reduction of DHS from baseline in response to air and cold stimulation at 2, 4, 6, and 8 weeks on a linear Visual Analog Scale (VAS). The study was conducted at the clinical research facility of the University of Texas Health San Antonio (UTHSA) School of Dentistry. The UTHSA Institutional Review Board (IRB) approved the study (protocol #HSC20150221H), and the study was conducted in accordance with the ethical standards outlined in the 1964 Declaration of Helsinki and its later amendments. The study was registered with ClinicalTrials.gov (NCT02918617). Written informed consent was obtained from all participants prior to their participation in the study. The majority of participants were recruited from among patients receiving treatment in the dental school clinic and from among employees of UTHSA.

### Participant recruitment

Subjects aged 18–80 years were given a screening examination that included a medical history, oral examination, and DHS evaluation of teeth with exposed buccal cervical dentin. DHS was assessed by a positive response to a 2-s blast of air from an air–water syringe.^1^ To be eligible for enrollment, the subject must have recorded a response of 20 mm on a 100-mm VAS on at least one tooth with exposed buccal cervical dentin.

Exclusion criteria were a history of adverse effects with the use of any oral hygiene products; use of over-the-counter desensitizing products within the previous 3 months, use of medications that could interfere with the perception of pain including chronic use of anti-inflammatory, analgesic, anticonvulsant, sedative, or other psychotropic drugs; and pregnancy or breastfeeding. Subjects were also excluded from participation if the sensitive tooth was associated with periodontal abscess, mobility >1, or pain from periodontal-related causes or occlusal trauma, or if the sensitive tooth was restored in the previous 3 months, or served as an abutment for a fixed or removable dental prosthesis.

### Washout period

Subjects who satisfied enrollment criteria were given a commercially available standard 1100 p.p.m. fluoride toothpaste (Colgate Cavity Protection, Colgate-Palmolive) and an adult soft-bristled toothbrush to use for a period of 1 week. Subjects were instructed to use the dentifrice and toothbrush for 2 min twice a day in place of their normally used dentifrice and toothbrush. The washout period is to allow for an attenuation of the residual effects of the subject’s previously used toothpaste, and to enable every subject to start on the same toothpaste background. Subjects were given no further instructions regarding oral hygiene and diet during the washout period.

### Study treatment

At the end of the washout period, patients returned to the clinic for baseline sensitivity measurements by the Clinical examiner (K.C.L. or S.S.). Ice-cold stimulus was applied using refrigerant spray (Endo-Ice, Hygenic) on a cotton pellet for 1 s. At least 10 min after ice-cold stimulus, air stimulus was applied using air delivered from a standard dental unit air syringe at room temperature (~70 ± 3 °F)(~21 ± 2 °C) and a pressure of 60 psi (±5 psi). The air was directed perpendicular to the exposed root surface of the sensitive tooth for 2 s from a distance of ~1 cm, and to prevent false results, the sensitive tooth was isolated from the adjacent teeth (mesial and distal) using cotton rolls held in place by the examiner’s gloved fingers. Patient response to ice-cold and air stimuli were recorded on the VAS for each sensitive tooth identified at the time of enrollment.

VAS was used within 2 min of stimulus application to evaluate the intensity of sensitivity on each measurement occasion. The VAS, which was well explained to the subjects, consists of an unmarked horizontal straight line, 100 mm long, drawn on a sheet of paper, with the beginning and end representing no pain and maximum pain, respectively. Following stimulus application as described above, the Clinical examiner requested the subject to draw a vertical mark on the line, indicating how much pain he/she just felt, with “no pain” being on the far left of the scale at 0 mm and “pain as bad as it can be,” i.e., the maximum pain the subject could experience, being on the far right at 100 mm. The distance between 0 mm and the mark made by the subject was measured in millimeters by the Clinical examiner and recorded as the subject’s VAS score, i.e., his/her indicated level of pain.

To ensure accurate interpretation and analysis of the sensitivity response, the primary (K.C.L.) and secondary (S.S.) examiners were trained and calibrated by a benchmark examiner (the principal investigator B.T.A.) on DHS assessment techniques. The first five subjects who were recruited into the study were used for the calibration exercise. These were patients who had been diagnosed as having DHS. The sensitivity evaluation data recorded during calibration comprised two measurements, Cold VAS and Air VAS. The agreement between the two examiners’ objective evaluation of sensitivity (interexaminer agreement) and each examiner’s individual sensitivity evaluation (intraexaminer agreement) was evaluated using the unweighted kappa (*κ*) statistic.

Using computer-generated randomization numbers from the biostatistics team, subjects were randomized by the study Randomizer (M.N.L.) to a group to use one of the following toothpaste formulations: toothpaste containing either 10% nano-HAP (10%nano-HAP), 15% nano-HAP (15%nano-HAP), 10% nano-HAP supplemented with 5% KNO_3_ (10%nano-HAPKN), or CSPS containing sodium monofluorophosphate 1.08% (w/w) (1450 p.p.m. fluoride). Study products’ description, chemical composition, chemical formula, and relative dentin abrasivity (RDA) are shown in Table 1. To ensure that patients and the research team, especially the Clinical examiner and the Recorder, were blinded as to product allocation, all toothpaste tubes were packaged identically but coded with the alphabet letters A through D by the manufacturing company, who retained the code until the completion of the study and data interpretation, thus blinding both the subjects and the investigators. Also, only the Randomizer was responsible for issuing the products, and was located in a cubicle separate from the clinical examination room.

**Table 1:** Study products description, chemical composition, chemical formula and relative dentin abrasivity (RDA).

Products description	RDA	Active ingredient	Chemical formula	Other ingredients
10% Nano-HAP toothpaste	80	10% Nano-hydroxyapatite	Ca_10_(PO_4_)_6_(OH)_2_	Cetylpyridinium chloride, β-glycyrrhetinic acid, dental-grade dibasic calcium phosphate, silicic anhydride, concentrated glycerin, water, sodium lauryl sulfate, sodium *N*-lauroyl sarcosinate, sodium carboxymethylcellulose, trimagnesium phosphate, saccharine sodium, alkyldiaminoethylglycine hydrochloride, flavor
15% Nano-HAP toothpaste	80	15% Nano-hydroxyapatite	Ca_10_(PO_4_)_6_(OH)_2_	Cetylpyridinium chloride, β-glycyrrhetinic acid, dental-grade dibasic calcium phosphate, silicic anhydride, concentrated glycerin, water, sodium lauryl sulfate, sodium *N*-lauroyl sarcosinate, sodium carboxymethylcellulose, trimagnesium phosphate, saccharine sodium, alkyldiaminoethylglycine hydrochloride, flavor
Nano-HAP supplemented with potassium nitrate (custom-made)	80	10% Nano-hydroxyapatite 5% Potassium nitrate	Ca_10_(PO_4_)_6_(OH)_2_ KNO_3_	Cetylpyridinium chloride, β-glycyrrhetinic acid, dental-grade dibasic calcium phosphate, silicic anhydride, concentrated glycerin, water, sodium lauryl sulfate, sodium *N*-lauroyl sarcosinate, sodium carboxymethylcellulose, trimagnesium phosphate, saccharine sodium, alkyldiaminoethylglycine hydrochloride, flavor
Sensodyne™ Repair & Protect with NovaMin^®^	104	15% Calcium sodium phosphosilicate (CSPS) Sodium monofluorophosphate 1.08% (w/w) (1450 p.p.m. fluoride)	CaO-Na_2_O-P_2_O_5_-SiO_2_ Na_2_PO_3_F	Glycerin, PEG-8, silica, cocamidopropyl betaine, sodium methyl cocoyl taurate, aroma, titanium dioxide, carbomer, sodium saccharin, limonene
Colgate Cavity Protection^®^ (regular)	68	Sodium monofluorophosphate 0.76% (0.15% (w/w) fluoride ion)	Na_2_PO_3_F	Dicalcium phosphate dehydrate, water, glycerin, sodium lauryl sulfate, celluse gum, flavor, tetrasodium pyrophosphate, sodium saccharin

Patients were instructed to continue brushing with their assigned toothpaste and toothbrush for 2 min in the morning and 2 min before bed, followed on each occasion by rinsing with 10 mL of water for 10 s. The first toothpaste-use occasion took place at the clinical research facility and was supervised by the study coordinator. Subjects were instructed not to take any food or drink for 30 min after toothpaste use. As a method of monitoring compliance, a calendar was provided to each subject for recording of daily toothpaste use, and in addition, toothpaste tubes were weighed at the time of randomization and at each study visit. Subjects were instructed to maintain their normal dietary habits, but were prohibited from using any other oral hygiene product (e.g., mouthwash, chewing gum) or tooth-whitening product for the duration of the study.

Subjects returned to the clinical research facility after 2, 4, 6, and 8 weeks. At each visit, response to ice-cold and air stimulus was recorded in two measurements (Cold VAS and Air VAS) as described above. Also at each visit, a clinical examiner screened the subjects for adverse events by questioning as well as examination of the subject’s oral cavity and perioral area.

### Power analysis and sample size calculation

The main outcomes were the difference across groups between the mean change in ice-cold and air stimuli test scores from baseline to the end of the study. The sample size calculations, which were based on a power analysis, were performed using nQuery Advisor software (Statistical Solutions). According to data from prior studies,^43,44^ and for the hypothesis that nano-HAP-containing toothpastes are as effective as a CSPS-containing toothpaste in relieving DHS, an effective sample size of 20 subjects in each treatment group would have statistical power >0.80 with a 0.05 one-sided significance level to detect a difference between a nano-HAP toothpaste and the CSPS toothpaste, and a sample mean percent change in VAS ≥10%. Using an unpaired *t* test and an expected withdrawal/dropout rate of 25%, a minimum of 25 subjects per group were required to achieve an 80% statistical power. The primary analysis was a linear mixed-effect model, which should achieve similar or greater power as the unpaired *t* test. The primary outcome examined in the present study was disease treatment, i.e., relief of DHS.

### Statistical methods

The normality distribution of all scores was assessed using the Shapiro–Wilk test. All subjects who entered the study from baseline completed the study per protocol; thus, per-protocol analysis was used. The VAS values were treated as continuous variables and assessed using a random-intercept, mixed-model analysis that accounted for the correlation among outcomes from the same patient. The primary test of the treatment effect was the treatment by time interaction. In addition, all the mean treatment outcomes at each time point were compared using the Duncan test for multiplicity with unequal sample sizes at 5% confidence interval. A *P* value of <0.05 was considered significant. Statistical analyses were conducted using the R environment for statistical computing (v3.1).

## Results

**Fig. 1:**
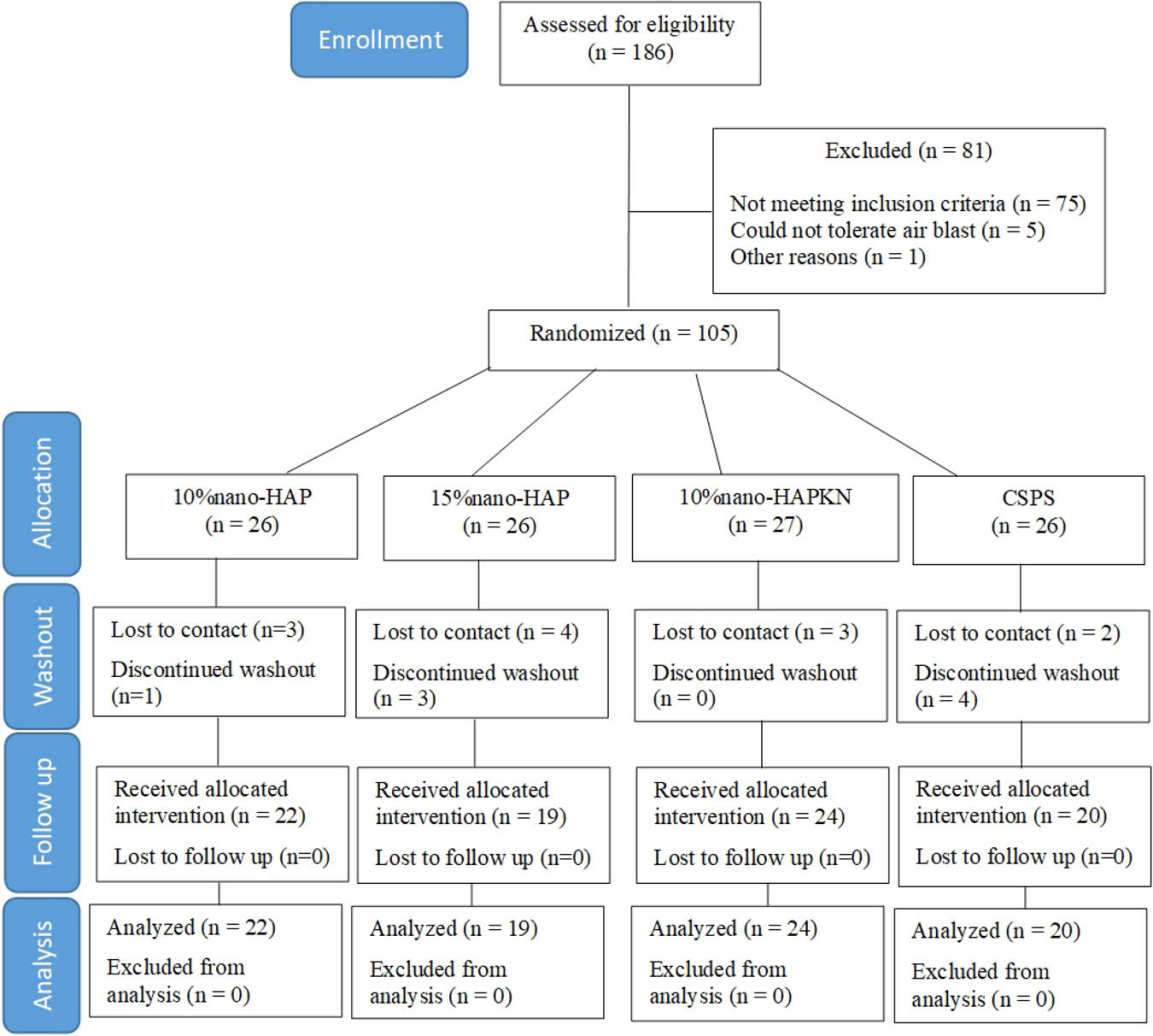
Patient flow diagram. CSPS calcium sodium phosphosilicate.

A flow diagram of participation in the trial is shown in Fig. 1. Of the 105 subjects recruited for this trial, 85 healthy adults (67 females and 18 males) with a mean (SD) age of 50.8 ± 11.4 years, from different ethnic origins and socioeconomic statuses, completed the study (Table 2). Twenty subjects dropped out from the study during the washout period; 12 were lost to contact (do not pick their calls), while 8 discontinued due to varying reasons (Fig. 1). Thus, all subjects who entered the study from baseline after the washout period completed the study per protocol. At no time was any incidence of adverse event or compromised oral health either reported by the subjects themselves or ascertained clinically.

**Table 2:** Subject demographics.

Test toothpaste	10%nano-HAP	15%nano-HAP	10%nano-HAPKN	CSPS	Total	*P* value*
Sex (%)						0.36
Female	16 (73%)	18 (95%)	18 (75%)	15 (75%)	67 (79%)	
Male	6 (27%)	1 (5%)	6 (25%)	5 (25%)	18 (21%)	
Total	22	19	24	20	85	
Age						0.26
Mean ± SD	52.8 ± 11.2	53.6 ± 11.0	47.3 ± 12.8	49.3 ± 10.5	50.8 ± 11.4	
Median [Q1, Q3]	55 [45, 60]	54 [48, 63]	49 [38, 58]	52 [47, 56]	52.5 [45, 59]	
Min, max	33, 71	32, 70	19, 64	24, 62	19, 71	
Ethnicity (%)						
American Indian/Alaskan Native	0 (0)	0 (0)	0 (0)	1 (5)	1 (1.18)	
Asian or Pacific Islander	1 (4.55)	0 (0)	0 (0)	3 (15)	4 (4.70)	
Black (not Hispanic)	2 (9.09)	1 (5.26)	0 (0)	1 (5)	4 (4.70)	
Hispanic	12 (54.55)	13 (68.42)	15 (62.5)	8 (40)	48 (56.47)	
Other	3 (13.64)	1 (5.26)	2 (8.33)	1 (5)	7 (8.24)	
White (not Hispanic)	4 (18.18)	4 (21.05)	7 (29.17)	6 (30)	21 (24.71)	
Total	22	19	24	20	85	

*CSPS* calcium sodium phosphosilicate.

*P value < 0.05.

The intraexaminer agreement values (unweighted *κ* value) for the two sensitivity measurements for the primary (K.C.L.) and secondary (S.S.) examiners, respectively. were 0.91/0.94 (Cold VAS) and 0.80/0.88 (Air VAS). The mean interexaminer reliability values for the two sensitivity measurements comparing the benchmark examiner versus each of the two examiners (K.C.L. and S.S.) were 0.72 (primary) and 0.80 (secondary). These values met the targets (0.70) set for acceptability prior to the training and calibration.

**Fig. 2:**
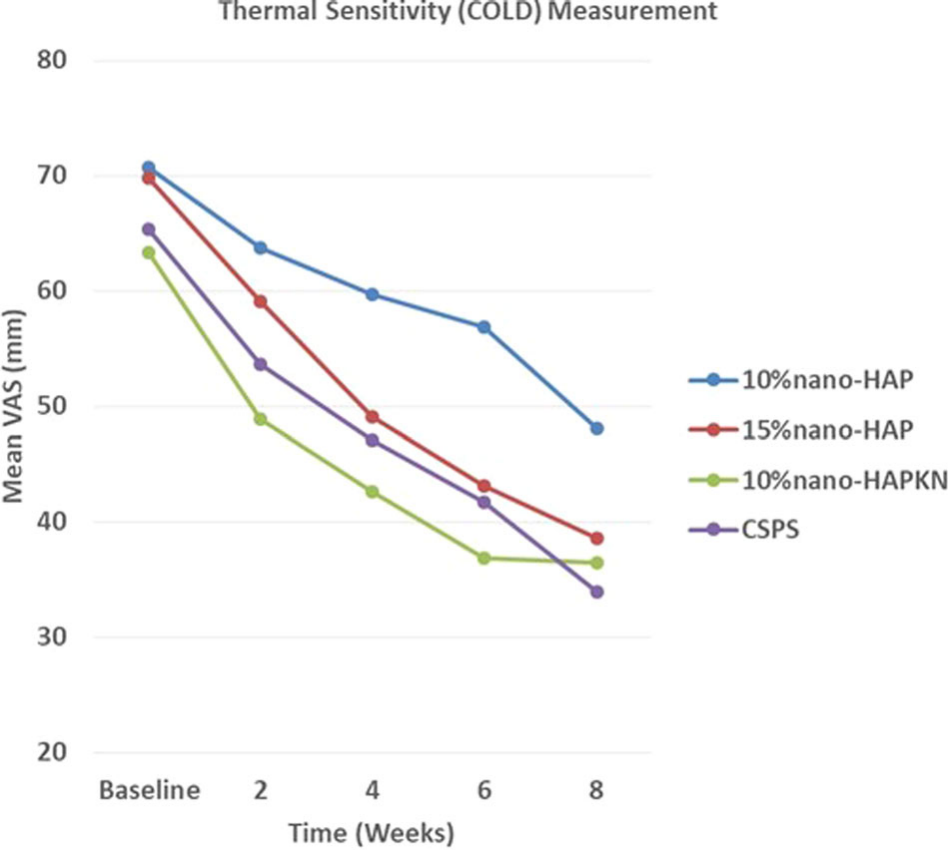
Reduction in DHS over time measured with VAS after a cold stimulus. For each product, there was a significant (*P* < 0.001) reduction in DHS at each time point. Comparing the reduction of DHS among the products, the following product pairs differed significantly (*P* < 0.001) from each other at the indicated measurement time period. Week 2: 10%nano-HAPKN and 10%nano-HAP; week 4: 10%nano-HAPKN and 10%nano-HAP; week 6: 10%nano-HAPKN and 10%nano-HAP, 15%nano-HAP and 10%nano-HAP, CSPS and 10%nano-HAP; and week 8: CSPS and 10%nano-HAP. CSPS calcium sodium phosphosilicate.

**Fig. 3:**
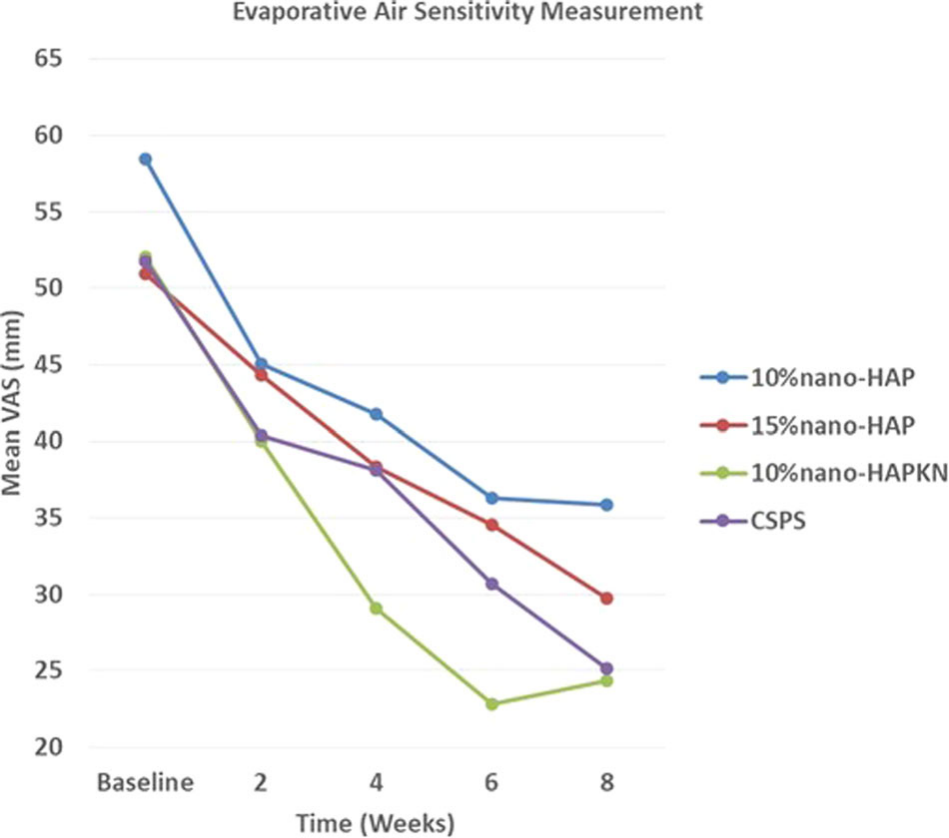
Reduction in DHS over time measured with VAS after evaporative air stimulus. For each product, there was a significant (*P* < 0.001) reduction in DHS at each time point. Comparing the reduction of DHS among the products, the following product pairs differed significantly (*P* < 0.001) from each other at the indicated measurement time period. Week 2: no significant difference among the products. Week 4: 10%nano-HAPKN and 10%nano-HAP; week 6: 10%nano-HAPKN and 10%nano-HAP, 10%nano-HAPKN and 15%nano-HAP; week 8: 10%nano-HAPKN and 10%nano-HAP, CSPS and 10%nano-HAP. CSPS calcium sodium phosphosilicate.

The reduction of DHS from baseline over time for the two sensitivity measurements is shown in Figs. 2 and 3. With either air or cold stimulus, VAS indicated significant (*P* < 0.001) reduction in DHS at each time point with all tested products. Also, with either air or cold stimulus, VAS showed 10%nano-HAPKN to have greater DHS reduction consistently when compared to the other nano-HAP-containing test toothpastes, and at most time points when compared with the positive control toothpaste containing CSPS. Among the nano-HAP-containing formulations, 15%nano-HAP and 10%nano-HAPKN were consistent in the reduction of DHS with all the two measurements (Air VAS and Cold VAS). With either air or cold stimulus, CSPS did not significantly differ with these two products (15%nano-HAP and 10%nano-HAPKN) at any time point with VAS measurement.

Comparing the reduction of DHS among the products using cold stimulus, there was a significantly greater reduction with 10%nano-HAPKN when compared with 10%nano-HAP at 2, 4, and 6 weeks, but not at 8 weeks measurement point. The reduction of sensitivity by CSPS was significantly greater at 6 and 8 weeks when compared with 10%nano-HAP. The difference between 10%nano-HAP- and 15%nano-HAP-containing toothpastes was only significant at 6 weeks, with greater sensitivity reduction with 15%nano-HAP.

Comparing the reduction of DHS among the products using evaporative air stimulus, there was no significant difference in the reduction of DHS among the products after 2 weeks of usage. However, at 4, 6, and 8 weeks, 10%nano-HAPKN made a significantly greater reduction in sensitivity when compared with 10%nano-HAP, but its sensitivity reduction was significantly greater than that of 15%nano-HAP only at 6 weeks. The reduction of sensitivity by CSPS was significantly greater (*P* < 0.001) than that of 10%nano-HAP only at 8 weeks (Figs. 2 and 3).

## Discussion

DHS is a condition prevalent in the adult population (4–74%),^4–6^ and commonly encountered in clinical dental practice, which has been shown to impact patient’s oral health-related quality of life by disturbing their eating, drinking, and toothbrushing as well as discouraging them from regular dental visits.^45,46^ Thus, the present clinical study investigated the effectiveness of three nano-HAP-containing toothpastes produced by Sangi Co., Ltd. of Japan to relieve DHS. The study compared the effectiveness of the nano-HAP toothpastes with that of a commercially available desensitizing toothpaste containing CSPS. Toothpaste formulations containing either 15%nano-HAP or 10%nano-HAPKN were as effective as the commercially available CSPS-containing toothpaste in reducing DHS at all measurement time points and with all applied sensitivity stimuli; thus, the result of the present study in the case of these toothpastes supported the study hypothesis that nano-HAP-containing toothpastes are as effective as a CSPS-containing toothpaste in relieving DHS (Figs. 2 and 3). However, in the case of the toothpaste containing 10%nano-HAP, the hypothesis was rejected with CSPS producing a significantly greater reduction of thermal sensitivity at 6 and 8 weeks and evaporative sensitivity at 8 weeks (Figs. 2 and 3). Nevertheless, the result of the present study is in agreement with several previous studies that demonstrated the efficacy in reducing DHS of nano-HAP applied as a toothpaste or in topical cream form.^42,43,47–50^ The effectiveness of nano-HAP to reduce DHS is attributed to its ability to form a layer of mineral hydroxyapatite as well as hydroxyapatite plugs that occlude dentinal tubules.^37–41^ The formation of a mineral hydroxyapatite layer and plugs is supported by the findings of previous studies that nano-HAP may act as a calcium and phosphate reservoir, helping to maintain a topical state of supersaturation of these ions with respect to tooth minerals, and thus causing mineral deposition on the surface of tooth tissue.^36,51^ Using a nano-HAP-containing toothpaste has been found to elevate calcium concentrations in saliva,^51^ and nano-HAP in toothpaste has also been reported to function by directly filling up micropores on tooth surfaces,^31^ and when it penetrates the pores in tooth tissues, it acts as a template in the apatite deposition process by continuously attracting large amounts of calcium and phosphate ions from the surrounding solutions (saliva, dentifrices, mouth rinses) to the tooth tissue; thus, promoting crystal integrity and growth.^31^ It is widely accepted that the pain in DHS results when external trigger stimuli cause rapid movement of the fluid within exposed dentinal tubules, which activates sensory nerve receptors within the pulp, resulting in the characteristic short, sharp pain of DHS.^8^ Thus, plugging of the exposed dentinal tubules by mineral hydroxyapatite, which has been shown to reduce the permeability of the tubules,^41^ would inevitably prevent the disturbance of the fluid within the tubules, and as such reduce DHS. The results of the present clinical study confirm that nano-HAP is a potent ingredient in toothpaste for decreasing DHS.

It was not surprising in the present study that supplementing 10%nano-HAP with KNO_3_ in a toothpaste resulted in a greater and consistent reduction in DHS, even greater (although not significantly different) than a well-established desensitizing toothpaste containing CSPS (Figs. 2 and 3). Previous studies have demonstrated that KNO_3_ enhances the reduction of DHS by nano-HAP.^38,52^ The efficacy of KNO_3_ in decreasing sensitivity has been well and long established.^53^ Potassium ions from the KNO_3_ diffuse along the dentinal tubules to modify the nerve impulse by depolarizing nerve fiber membranes and rendering them unable to repolarize, resulting in a reduction in tooth sensitivity by rendering the nerves unresponsive to excitatory stimuli; however, the effect of KNO_3_ alone is cumulative and it may take several weeks for patients to feel any pain reduction.^54^ Nonetheless, when nano-HAP is supplemented with KNO_3_, as in the case in the present study, the KNO_3_ works on the nerve receptors, while nano-HAP occludes the dentinal tubules to limit the movement of the dentinal fluid, resulting in an enhanced efficacy in reducing DHS as was demonstrated in the present study. Thus, although toothpaste containing 10%nano-HAP was not as effective as the other nano-HAP toothpastes and CSPS toothpaste in the present study, results showed that supplementing 10%nano-HAP with KNO_3_ enhanced the efficacy of the 10%nano-HAP in reducing DHS. However, the results in Figs. 2 (cold stimulus) and 3 (air stimulus) both show that the efficacy of the 10%nano-HAPKN toothpaste stagnates and even diminishes after 6 weeks of application, in contrast to all other products tested. This may be attributed to nano-HAP blocking the diffusion of KNO_3_ into the tubules after longer periods of application.

Although there was no statistically significant difference in sensitivity reduction between the toothpastes containing 10 and 15% nano-HAP toothpaste at any measurement time point (except at 6 weeks with cold sensitivity), quantitatively 15%nano-HAP caused a greater reduction in both cold sensitivity and evaporative air sensitivity than the 10%nano-HAP toothpaste at every time point (Figs. 2 and 3). This observation can be attributed to the finding of a previous in situ study, which compared the dentinal tubule occlusion of 10 and 15%nano-HAP, and found that although increasing the concentration of nano-HAP from 10 to 15% in the dentifrice did not produce any significant difference in its tubule occlusion and mineral layer deposition effectiveness; nevertheless, a thicker layer of mineral hydroxyapatite deposit with 15% concentration was observed.^41^ Furthermore, the findings of the present study are also in agreement with those of a previous clinical study that compared the sensitivity reduction of different nano-HAP concentrations, and suggested that the higher concentration probably enhanced better penetration of the particles into the tubules, based on the observation that most of the patients in the study who used higher concentration obtained relief with just one or two applications of hydroxyapatite.^55^ However, one may not rule out the fact that the sample size in this study may be too small to show a statistical difference (Type II Error) between the two concentrations.

In the present study, a commercially available toothpaste containing silica-based bioglass, namely CSPS, was used as the standard for DHS reduction, considering that there are several research reports supporting the effectiveness of this device to reduce DHS.^20–23,50,56^ The mode of action of this material is based on its chemical reactivity with aqueous solutions. When introduced into the oral environment, the material reportedly releases sodium, calcium, and phosphate, which then interact with the oral fluids and result in the formation of a crystalline hydroxycarbonate apatite layer that is structurally and chemically similar to natural tooth mineral.^57^ With this mode of action, it was expected that CSPS may perform significantly better than the nano-HAP devices; however, the calcium and phosphate ions in CSPS are protected by glass and the glass particles need to be trapped for the calcium and phosphate to be localized, and this may cause a delay in the action of CSPS,^58^ which may explain why the effectiveness of the CSPS toothpaste in reducing DHS did not differ significantly from that of nano-HAP-containing toothpastes. Nevertheless, the fact that these three agents, 15%nano-HAP, 10%nano-HAPKN, and CSPS, exhibited equal effectiveness in reducing DHS, they can serve as an alternative to each other to be used by adults suffering from DHS.

Although we believed the subjects recruited into the study were well motivated to participate in the study, we experienced an unexpected high number of dropouts (≈20%), all of which occurred at the washout period. The major reason given by the subjects, who informed the research team of their withdrawal, was unwilling to continue with the stimuli applications, especially the air blast. Other dropouts stopped picking calls from the research team, and we believed they may share the same reason. The washout period was to allow for an attenuation of the residual effect of the subject’s previously used toothpaste, and to enable every subject to start on the same background. Furthermore, one may wonder why the mean value of the baseline sensitivity scores varied among the groups, e.g., the 10%nano-HAP had the highest DHS baseline score. This is because the randomization of the subjects was based on their age to ensure there is no significant difference in age between the groups due to the reported variation in DHS prevalence between age groups.^5^ However, we must admit that stratification based on the baseline DHS values is more important than the age, as it could potentially influence the results.

## Conclusion

Within the limits of this study, it can be concluded that toothpaste containing nano-HAP alone (10 or 15% nano-HAP) or supplemented with KNO_3_ (10%nano-HAKN) was effective in relieving DHS symptoms when used at least twice daily. The study further demonstrated that the toothpaste containing 15% nano-HAP was more effective in sensitivity reduction than that containing 10% nano-HAP.
